# 2-[2-(1*H*-Imidazol-1-yl)-2-adamant­yl]phenol

**DOI:** 10.1107/S1600536810053900

**Published:** 2011-01-15

**Authors:** Vitalij A. Osyanin, Yulia V. Popova, Victor B. Rybakov, Yurij N. Klimochkin

**Affiliations:** a Samara State Technical University, Molodogvardeyskay Str. 244, 443100 Samara, Russian Federation; b Department of Chemistry, Moscow State University, 119992 Moscow, Russian Federation

## Abstract

In the title mol­ecule, C_19_H_22_N_2_O, the imidazole and benzene rings form a dihedral angle of 84.53 (5)°. In the crystal, classical inter­molecular O—H⋯N hydrogen bonds pair the mol­ecules into centrosymmetric dimers, and C—H⋯π inter­actions further link these dimers into columns propagated in [100].

## Related literature

For the role of *o*-quinone methides in the biological action of several anti­biotics such as mitomycin and anthracyclines, see: Rokita (2009 ▶). For the reaction mechanism, see: Van De Water & Pettus (2002 ▶).
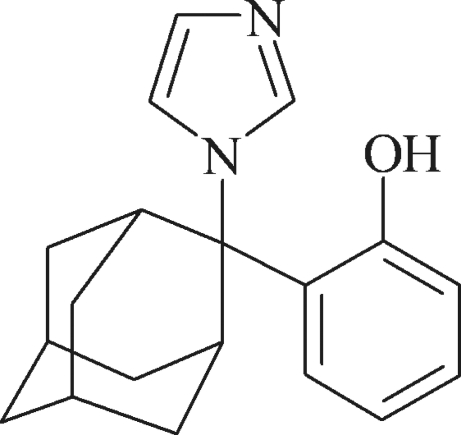


## Experimental

### 

#### Crystal data

C_19_H_22_N_2_O
*M* *_r_* = 294.39
Triclinic, 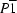

*a* = 6.3981 (4) Å
*b* = 10.3944 (7) Å
*c* = 12.5345 (8) Å
α = 67.028 (6)°
β = 84.863 (6)°
γ = 72.647 (6)°
*V* = 732.22 (9) Å^3^
*Z* = 2
Cu *K*α radiation
μ = 0.65 mm^−1^
*T* = 100 K
0.56 × 0.19 × 0.12 mm

#### Data collection

Oxford Diffraction Xcalibur Atlas Gemini ultra diffractometer
Absorption correction: analytical [*CrysAlis PRO RED* (Oxford Diffraction, 2010 ▶); based on expressions derived by Clark & Reid (1995 ▶)] *T*_min_ = 0.819, *T*_max_ = 0.944
6557 measured reflections
2600 independent reflections
2300 reflections with *I* > 2σ(*I*)
*R*_int_ = 0.024

#### Refinement

*R*[*F* ^2^ > 2σ(*F* ^2^)] = 0.039
*wR*(*F* ^2^) = 0.106
*S* = 1.05
2600 reflections
200 parameters
H-atom parameters constrained
Δρ_max_ = 0.28 e Å^−3^
Δρ_min_ = −0.27 e Å^−3^

### 

Data collection: *CrysAlis PRO CCD* (Oxford Diffraction, 2010 ▶); cell refinement: *CrysAlis PRO CCD*; data reduction: *CrysAlis PRO RED* (Oxford Diffraction, 2010 ▶); program(s) used to solve structure: *OLEX2* (Dolomanov *et al.*, 2009 ▶); program(s) used to refine structure: *SHELXL97* (Sheldrick, 2008 ▶); molecular graphics: *ORTEP-3* (Farrugia, 1997 ▶); software used to prepare material for publication: *OLEX2*.

## Supplementary Material

Crystal structure: contains datablocks global, I. DOI: 10.1107/S1600536810053900/cv5021sup1.cif

Structure factors: contains datablocks I. DOI: 10.1107/S1600536810053900/cv5021Isup2.hkl

Additional supplementary materials:  crystallographic information; 3D view; checkCIF report

## Figures and Tables

**Table 1:** Hydrogen-bond geometry (Å, °) *Cg* is the centroid of the C11–C16 ring.

*D*—H⋯*A*	*D*—H	H⋯*A*	*D*⋯*A*	*D*—H⋯*A*
O16—H16⋯N19^i^	0.84	1.83	2.6514 (15)	164
C20—H20⋯*Cg*^ii^	0.95	2.60	3.459 (18)	151

Symmetry codes: (i) 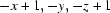; (ii) 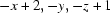.

**Fig. 1.**
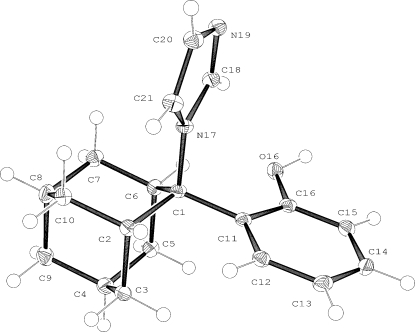
ORTEP-3 (Farrugia, 1997) plot of molecular structure of the title compound showing the atom-numbering scheme. Displacement ellipsoids are drawn at the 50% probability level.

**Fig. 2.**
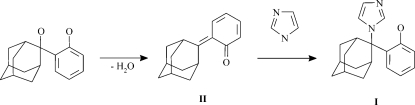
Synthesis of the title compound.

## supplementary crystallographic information

### Comment

*o*-Quinone methides are known as efficient *DNA* alkylating and cross-linking agents, they play a key role in the biological action of several antibiotics such as mitomycin and anthracyclines (Rokita, 2009). *o*-Quinone methides act as heterodienes in inter- and intramolecular cycloadditions with olefins to give various substituted chromanes. Like vinyl ketones, *o*-quinone methides also act as acceptors in Michael additions to afford *o*-substituted phenols (Van De Water & Pettus, 2002). Herewith we presenrt the title compound, I, which belongs to the family of *o*-quinone methides.

In I (Fig. 1), benzene ring is essential planar and hydroxy O deviates at 0.0171 (18) Å) from its mean plane. The benzene and imidazole rings form dihedral angle 84.53 (5)°.

In the crystal structure, intermolecular O–H···N hydrogen bonds (Table 1) pair the molecules into centrosymmetric dimers, and C–H···π interactions (Table 1) link further these dimers into columns propagated in direction [1 0 0].

### Experimental

2-[2-(1*H*-Imidazol-1-yl)-2-adamantyl]phenol, I, was prepared from 2-(2-hydroxyphenyl)-2-adamantanol and imidazole in *DMF* at reflux in 74% yield. A mechanism accounting for the formation of structure I is depicted in Fig. 2. The 2-(2-hydroxyphenyl)-2-adamantanol loses a molecule of water to give the *o*-quinone methide II. A Michael-type addition of the imidazole to the *o*-quinone methide II gives the end product I.

A solution of 2-(2-hydroxyphenyl)-2-adamantanol (1 g, 4.1 mmol) and imidazole (1 g, 14.7 mmol) in *DMF* (10 ml) was refluxed for 2 h. After completion of the reaction, the mixture was cooled to room temperature, poured into 30 ml of cold water to yield a solid product, which was filtered, washed with water, and dried. Recrystallization of the crude product from ethanol gave 0.89 g of colourless crystals. Yield 74%, mp 524-525 K. IR, ν, cm^-1^: 3200-2400 (OH), 2920, 2858 (CH*Ad*), 1597, 1493, 1450, 1404, 1296, 1250, 1234, 1219, 1204, 1111, 1095, 1072, 752, 663. MS, m/*z*: 294 [*M*]^+^ (9), 226 [C_16_H_18_O]^+^ (100), 211 (8), 183 (46), 169 (26), 158 (19), 145 (17), 131 (24), 115 (23), 107 (22), 91 (26), 79 (20), 77 (24), 69 (36). ^1^H NMR, δ: 1.72-1.81 m (11*H*, H*Ad*), 2.00 br. s (1*H*, H*Ad*), 3.35 br. s and 4.17 br. s (2*H*, H*Ad*-1,3), 6.71-6.78 m (3*H*, H_arom._-4,6, H_imidazole_-4), 6.99 dd (1*H*, H_arom._-5, ^3^J = 8.07 Hz, ^3^J = 7.34 Hz), 7.24 s (1*H*, H_imidazole_-5), 7.48 d (1*H*, H_arom._-3, ^3^J = 7.34 Hz), 7.80 s (1*H*, H_imidazole_-2), 9.46 br. s (1*H*, OH). Anal. calc. for C_19_H_22_N_2_O, %: C 77.52; H 7.53; N 9.52. Found, %: C 77.59; H 7.48; N 9.48.

Single crystals for *X*-ray analysis were obtained by slow evaporation of an ethanol solution. IR-spectrum was recorded (in KBr) on Shimadzu FTIR-8400S. Mass-spectrum was measured on Finnigan Trance DSQ spectrometer. ^1^H NMR-spectrum was obtained in *DMSO*-*d*_6_ on Jeol JNM-ECX400 (400 MHz), using *TMS* as internal standard. Elemental composition was determined on Euro Vector EA-3000 elemental analyzer.

### Refinement

All H atoms were placed in calculated positions (C–H 0.95-1.00Å and O–H 0.84 Å) and refined as riding, with *U*_iso_(H) = 1.2-1.5 *U*_eq_(C, O).

### Crystal data

**Table:**

C_19_H_22_N_2_O	*Z* = 2
*M**_r_* = 294.39	*F*(000) = 316
Triclinic, *P*1	*D*_x_ = 1.335 Mg m^−^^3^
Hall symbol: -P 1	Melting point = 524–525 K
*a* = 6.3981 (4) Å	Cu *K*α radiation, λ = 1.5418 Å
*b* = 10.3944 (7) Å	Cell parameters from 4464 reflections
*c* = 12.5345 (8) Å	θ = 3.8–67.4°
α = 67.028 (6)°	µ = 0.65 mm^−^^1^
β = 84.863 (6)°	*T* = 100 K
γ = 72.647 (6)°	Prism, colourless
*V* = 732.22 (9) Å^3^	0.56 × 0.19 × 0.12 mm

### Data collection

**Table:**

Oxford Diffraction Xcalibur Atlas Gemini ultra diffractometer	2600 independent reflections
Radiation source: fine-focus sealed tube	2300 reflections with *I* > 2σ(*I*)
mirror	*R*_int_ = 0.024
Detector resolution: 10.4875 pixels mm^-1^	θ_max_ = 67.5°, θ_min_ = 3.8°
ω scans	*h* = −7→7
Absorption correction: analytical [*CrysAlis PRO RED* (Oxford Diffraction, 2010); based on expressions derived by Clark & Reid (1995)]	*k* = −12→11
*T*_min_ = 0.819, *T*_max_ = 0.944	*l* = −14→14
6557 measured reflections	

### Refinement

**Table:**

Refinement on *F*^2^	Primary atom site location: structure-invariant direct methods
Least-squares matrix: full	Secondary atom site location: difference Fourier map
*R*[*F*^2^ > 2σ(*F*^2^)] = 0.039	Hydrogen site location: inferred from neighbouring sites
*wR*(*F*^2^) = 0.106	H-atom parameters constrained
*S* = 1.05	*w* = 1/[σ^2^(*F*_o_^2^) + (0.0569*P*)^2^ + 0.2906*P*] where *P* = (*F*_o_^2^ + 2*F*_c_^2^)/3
2600 reflections	(Δ/σ)_max_ < 0.001
200 parameters	Δρ_max_ = 0.28 e Å^−^^3^
0 restraints	Δρ_min_ = −0.27 e Å^−^^3^

### Special details

**Table:**

Experimental. *CrysAlis Pro Red*. Analytical numeric absorption correction using a multifaceted crystal model based on expressions derived by Clark & Reid (1995).
Geometry. All s.u.'s (except the s.u. in the dihedral angle between two l.s. planes) are estimated using the full covariance matrix. The cell s.u.'s are taken into account individually in the estimation of s.u.'s in distances, angles and torsion angles; correlations between s.u.'s in cell parameters are only used when they are defined by crystal symmetry. An approximate (isotropic) treatment of cell s.u.'s is used for estimating s.u.'s involving l.s. planes.
Refinement. Refinement of F^2^ against ALL reflections. The weighted R-factor wR and goodness of fit S are based on F^2^, conventional R-factors R are based on F, with F set to zero for negative F^2^. The threshold expression of F^2^ > 2σ(F^2^) is used only for calculating R-factors(gt) etc. and is not relevant to the choice of reflections for refinement. R-factors based on F^2^ are statistically about twice as large as those based on F, and R-factors based on ALL data will be even larger.

### Fractional atomic coordinates and isotropic or equivalent isotropic displacement parameters (Å^2^)

**Table:**

	*x*	*y*	*z*	*U*_iso_*/*U*_eq_	
C1	0.6828 (2)	0.13046 (14)	0.71608 (11)	0.0119 (3)	
C2	0.8219 (2)	0.19052 (14)	0.77095 (11)	0.0129 (3)	
H2	0.9810	0.1423	0.7659	0.015*	
C3	0.7648 (2)	0.15615 (15)	0.89927 (11)	0.0144 (3)	
H3A	0.7957	0.0494	0.9413	0.017*	
H3B	0.8567	0.1909	0.9352	0.017*	
C4	0.5221 (2)	0.23102 (15)	0.90889 (12)	0.0159 (3)	
H4	0.4851	0.2063	0.9924	0.019*	
C5	0.3829 (2)	0.17734 (15)	0.85153 (12)	0.0150 (3)	
H5B	0.2256	0.2242	0.8580	0.018*	
H5A	0.4112	0.0705	0.8919	0.018*	
C6	0.4385 (2)	0.21431 (14)	0.72319 (11)	0.0133 (3)	
H6	0.3426	0.1818	0.6864	0.016*	
C7	0.3972 (2)	0.38008 (14)	0.66138 (12)	0.0158 (3)	
H7A	0.4352	0.4041	0.5789	0.019*	
H7B	0.2399	0.4299	0.6645	0.019*	
C8	0.5352 (2)	0.43437 (15)	0.71904 (12)	0.0168 (3)	
H8	0.5069	0.5423	0.6784	0.020*	
C9	0.4767 (2)	0.39671 (15)	0.84690 (12)	0.0183 (3)	
H9A	0.5652	0.4319	0.8844	0.022*	
H9B	0.3200	0.4456	0.8527	0.022*	
C10	0.7765 (2)	0.35711 (14)	0.70967 (12)	0.0157 (3)	
H10B	0.8701	0.3907	0.7456	0.019*	
H10A	0.8134	0.3822	0.6270	0.019*	
C11	0.7345 (2)	−0.03789 (14)	0.77320 (11)	0.0121 (3)	
C12	0.9364 (2)	−0.12251 (15)	0.83113 (11)	0.0142 (3)	
H12	1.0346	−0.0742	0.8399	0.017*	
C13	0.9993 (2)	−0.27371 (15)	0.87630 (12)	0.0170 (3)	
H13	1.1375	−0.3271	0.9153	0.020*	
C14	0.8589 (2)	−0.34664 (15)	0.86411 (12)	0.0173 (3)	
H14	0.9000	−0.4502	0.8949	0.021*	
C15	0.6586 (2)	−0.26690 (15)	0.80662 (11)	0.0158 (3)	
H15	0.5624	−0.3167	0.7981	0.019*	
C16	0.5952 (2)	−0.11438 (15)	0.76088 (11)	0.0135 (3)	
O16	0.39755 (15)	−0.04140 (10)	0.70444 (8)	0.0160 (2)	
H16	0.3581	−0.0954	0.6799	0.024*	
N17	0.73914 (18)	0.16304 (12)	0.59155 (9)	0.0128 (3)	
C18	0.6190 (2)	0.15886 (14)	0.50968 (11)	0.0149 (3)	
H18	0.4767	0.1459	0.5218	0.018*	
N19	0.72161 (19)	0.17480 (12)	0.41173 (10)	0.0160 (3)	
C20	0.9204 (2)	0.18904 (15)	0.43073 (12)	0.0164 (3)	
H20	1.0312	0.2028	0.3751	0.020*	
C21	0.9346 (2)	0.18058 (15)	0.54089 (12)	0.0155 (3)	
H21	1.0556	0.1857	0.5762	0.019*	

### Atomic displacement parameters (Å^2^)

**Table:**

	*U*^11^	*U*^22^	*U*^33^	*U*^12^	*U*^13^	*U*^23^
C1	0.0118 (6)	0.0129 (7)	0.0096 (6)	−0.0018 (5)	0.0004 (5)	−0.0043 (5)
C2	0.0111 (6)	0.0135 (7)	0.0136 (7)	−0.0021 (5)	−0.0007 (5)	−0.0056 (5)
C3	0.0162 (7)	0.0144 (6)	0.0124 (7)	−0.0028 (5)	−0.0013 (5)	−0.0059 (5)
C4	0.0174 (7)	0.0160 (7)	0.0140 (6)	−0.0030 (5)	0.0023 (5)	−0.0072 (5)
C5	0.0128 (6)	0.0142 (7)	0.0161 (7)	−0.0016 (5)	0.0024 (5)	−0.0060 (5)
C6	0.0110 (6)	0.0125 (7)	0.0146 (7)	−0.0006 (5)	−0.0008 (5)	−0.0053 (5)
C7	0.0138 (6)	0.0135 (7)	0.0164 (7)	−0.0002 (5)	−0.0011 (5)	−0.0042 (5)
C8	0.0179 (7)	0.0112 (6)	0.0191 (7)	−0.0017 (5)	−0.0007 (5)	−0.0051 (5)
C9	0.0186 (7)	0.0163 (7)	0.0207 (7)	−0.0015 (5)	0.0010 (6)	−0.0104 (6)
C10	0.0173 (7)	0.0150 (7)	0.0153 (7)	−0.0055 (5)	0.0003 (5)	−0.0058 (5)
C11	0.0125 (6)	0.0136 (7)	0.0090 (6)	−0.0013 (5)	0.0022 (5)	−0.0053 (5)
C12	0.0142 (6)	0.0156 (7)	0.0126 (6)	−0.0024 (5)	0.0004 (5)	−0.0067 (5)
C13	0.0163 (7)	0.0164 (7)	0.0134 (6)	0.0023 (5)	−0.0021 (5)	−0.0050 (5)
C14	0.0244 (7)	0.0114 (6)	0.0134 (6)	−0.0017 (5)	0.0011 (5)	−0.0043 (5)
C15	0.0193 (7)	0.0161 (7)	0.0137 (6)	−0.0063 (5)	0.0026 (5)	−0.0071 (5)
C16	0.0135 (6)	0.0162 (7)	0.0097 (6)	−0.0018 (5)	0.0020 (5)	−0.0059 (5)
O16	0.0148 (5)	0.0157 (5)	0.0185 (5)	−0.0033 (4)	−0.0032 (4)	−0.0076 (4)
N17	0.0130 (6)	0.0138 (6)	0.0102 (5)	−0.0021 (4)	−0.0005 (4)	−0.0042 (4)
C18	0.0148 (6)	0.0144 (7)	0.0139 (7)	−0.0027 (5)	−0.0019 (5)	−0.0043 (5)
N19	0.0179 (6)	0.0146 (6)	0.0137 (6)	−0.0024 (5)	−0.0009 (5)	−0.0050 (4)
C20	0.0154 (7)	0.0172 (7)	0.0149 (7)	−0.0028 (5)	0.0020 (5)	−0.0060 (5)
C21	0.0122 (6)	0.0192 (7)	0.0151 (7)	−0.0033 (5)	0.0012 (5)	−0.0075 (5)

### Geometric parameters (Å, °)

**Table:**

C1—N17	1.4968 (16)	C9—H9A	0.9900
C1—C11	1.5503 (18)	C9—H9B	0.9900
C1—C2	1.5591 (18)	C10—H10B	0.9900
C1—C6	1.5600 (17)	C10—H10A	0.9900
C2—C3	1.5402 (18)	C11—C12	1.4006 (19)
C2—C10	1.5410 (18)	C11—C16	1.4121 (19)
C2—H2	1.0000	C12—C13	1.386 (2)
C3—C4	1.5329 (19)	C12—H12	0.9500
C3—H3A	0.9900	C13—C14	1.387 (2)
C3—H3B	0.9900	C13—H13	0.9500
C4—C5	1.5317 (19)	C14—C15	1.384 (2)
C4—C9	1.5335 (19)	C14—H14	0.9500
C4—H4	1.0000	C15—C16	1.3983 (19)
C5—C6	1.5358 (18)	C15—H15	0.9500
C5—H5B	0.9900	C16—O16	1.3578 (17)
C5—H5A	0.9900	O16—H16	0.8400
C6—C7	1.5369 (18)	N17—C18	1.3572 (18)
C6—H6	1.0000	N17—C21	1.3800 (18)
C7—C8	1.5334 (19)	C18—N19	1.3144 (18)
C7—H7A	0.9900	C18—H18	0.9500
C7—H7B	0.9900	N19—C20	1.3750 (18)
C8—C10	1.5307 (19)	C20—C21	1.359 (2)
C8—C9	1.5336 (19)	C20—H20	0.9500
C8—H8	1.0000	C21—H21	0.9500
			
N17—C1—C11	105.20 (10)	C7—C8—H8	109.9
N17—C1—C2	109.43 (10)	C9—C8—H8	109.9
C11—C1—C2	112.54 (10)	C4—C9—C8	109.60 (11)
N17—C1—C6	109.23 (10)	C4—C9—H9A	109.8
C11—C1—C6	114.20 (10)	C8—C9—H9A	109.8
C2—C1—C6	106.20 (10)	C4—C9—H9B	109.8
C3—C2—C10	107.87 (10)	C8—C9—H9B	109.8
C3—C2—C1	109.39 (10)	H9A—C9—H9B	108.2
C10—C2—C1	111.95 (10)	C8—C10—C2	110.44 (11)
C3—C2—H2	109.2	C8—C10—H10B	109.6
C10—C2—H2	109.2	C2—C10—H10B	109.6
C1—C2—H2	109.2	C8—C10—H10A	109.6
C4—C3—C2	110.01 (11)	C2—C10—H10A	109.6
C4—C3—H3A	109.7	H10B—C10—H10A	108.1
C2—C3—H3A	109.7	C12—C11—C16	116.59 (12)
C4—C3—H3B	109.7	C12—C11—C1	120.07 (11)
C2—C3—H3B	109.7	C16—C11—C1	123.06 (11)
H3A—C3—H3B	108.2	C13—C12—C11	122.87 (13)
C5—C4—C3	108.87 (11)	C13—C12—H12	118.6
C5—C4—C9	109.55 (11)	C11—C12—H12	118.6
C3—C4—C9	109.45 (11)	C12—C13—C14	119.57 (13)
C5—C4—H4	109.6	C12—C13—H13	120.2
C3—C4—H4	109.6	C14—C13—H13	120.2
C9—C4—H4	109.6	C15—C14—C13	119.31 (13)
C4—C5—C6	110.18 (11)	C15—C14—H14	120.3
C4—C5—H5B	109.6	C13—C14—H14	120.3
C6—C5—H5B	109.6	C14—C15—C16	121.17 (13)
C4—C5—H5A	109.6	C14—C15—H15	119.4
C6—C5—H5A	109.6	C16—C15—H15	119.4
H5B—C5—H5A	108.1	O16—C16—C15	118.79 (12)
C5—C6—C7	109.36 (11)	O16—C16—C11	120.71 (12)
C5—C6—C1	108.42 (10)	C15—C16—C11	120.49 (12)
C7—C6—C1	111.46 (11)	C16—O16—H16	109.5
C5—C6—H6	109.2	C18—N17—C21	105.78 (11)
C7—C6—H6	109.2	C18—N17—C1	125.96 (11)
C1—C6—H6	109.2	C21—N17—C1	127.56 (11)
C8—C7—C6	110.49 (11)	N19—C18—N17	112.41 (12)
C8—C7—H7A	109.6	N19—C18—H18	123.8
C6—C7—H7A	109.6	N17—C18—H18	123.8
C8—C7—H7B	109.6	C18—N19—C20	105.33 (11)
C6—C7—H7B	109.6	C21—C20—N19	109.81 (12)
H7A—C7—H7B	108.1	C21—C20—H20	125.1
C10—C8—C7	107.65 (11)	N19—C20—H20	125.1
C10—C8—C9	109.96 (11)	C20—C21—N17	106.65 (12)
C7—C8—C9	109.52 (11)	C20—C21—H21	126.7
C10—C8—H8	109.9	N17—C21—H21	126.7
			
N17—C1—C2—C3	−179.67 (10)	C1—C2—C10—C8	60.58 (14)
C11—C1—C2—C3	−63.10 (13)	N17—C1—C11—C12	95.89 (13)
C6—C1—C2—C3	62.54 (13)	C2—C1—C11—C12	−23.18 (16)
N17—C1—C2—C10	60.82 (13)	C6—C1—C11—C12	−144.35 (12)
C11—C1—C2—C10	177.39 (10)	N17—C1—C11—C16	−77.81 (14)
C6—C1—C2—C10	−56.97 (13)	C2—C1—C11—C16	163.12 (11)
C10—C2—C3—C4	60.61 (13)	C6—C1—C11—C16	41.95 (17)
C1—C2—C3—C4	−61.39 (13)	C16—C11—C12—C13	−0.85 (19)
C2—C3—C4—C5	58.63 (14)	C1—C11—C12—C13	−174.96 (12)
C2—C3—C4—C9	−61.09 (14)	C11—C12—C13—C14	0.3 (2)
C3—C4—C5—C6	−59.90 (14)	C12—C13—C14—C15	0.2 (2)
C9—C4—C5—C6	59.76 (14)	C13—C14—C15—C16	−0.1 (2)
C4—C5—C6—C7	−58.55 (13)	C14—C15—C16—O16	179.48 (12)
C4—C5—C6—C1	63.17 (13)	C14—C15—C16—C11	−0.5 (2)
N17—C1—C6—C5	179.01 (10)	C12—C11—C16—O16	−179.02 (11)
C11—C1—C6—C5	61.54 (14)	C1—C11—C16—O16	−5.11 (19)
C2—C1—C6—C5	−63.08 (13)	C12—C11—C16—C15	0.93 (19)
N17—C1—C6—C7	−60.57 (13)	C1—C11—C16—C15	174.84 (11)
C11—C1—C6—C7	−178.04 (10)	C11—C1—N17—C18	75.31 (15)
C2—C1—C6—C7	57.34 (13)	C2—C1—N17—C18	−163.57 (12)
C5—C6—C7—C8	58.29 (14)	C6—C1—N17—C18	−47.69 (16)
C1—C6—C7—C8	−61.58 (14)	C11—C1—N17—C21	−93.75 (14)
C6—C7—C8—C10	60.48 (14)	C2—C1—N17—C21	27.38 (17)
C6—C7—C8—C9	−59.05 (14)	C6—C1—N17—C21	143.25 (12)
C5—C4—C9—C8	−60.07 (14)	C21—N17—C18—N19	−1.09 (15)
C3—C4—C9—C8	59.24 (14)	C1—N17—C18—N19	−172.09 (11)
C10—C8—C9—C4	−58.49 (14)	N17—C18—N19—C20	0.49 (15)
C7—C8—C9—C4	59.61 (14)	C18—N19—C20—C21	0.33 (15)
C7—C8—C10—C2	−59.76 (14)	N19—C20—C21—N17	−0.98 (15)
C9—C8—C10—C2	59.49 (14)	C18—N17—C21—C20	1.22 (14)
C3—C2—C10—C8	−59.81 (14)	C1—N17—C21—C20	172.03 (12)

### Hydrogen-bond geometry (Å, °)

**Table:**

Cg is the centroid of the C11–C16 ring.

**Table:**

*D*—H···*A*	*D*—H	H···*A*	*D*···*A*	*D*—H···*A*
O16—H16···N19^i^	0.84	1.83	2.6514 (15)	164
C20—H20···Cg^ii^	0.95	2.60	3.459 (18)	151

Symmetry codes: (i) −*x*+1, −*y*, −*z*+1; (ii) −*x*+2, −*y*, −*z*+1.
